# Supplementary material for: Room-temperature low-threshold avalanche effect in stepwise van-der-Waals homojunction photodiodes
Source: Nat Commun. 2024 Apr 29;15:3639. doi: 10.1038/s41467-024-47958-2 (PMC11059283; doi:10.1038/s41467-024-47958-2)
Supplement: Supplementary file 1 — Supplementary Information [file 41467_2024_47958_MOESM1_ESM.pdf]

Supplementary Information for

Room-temperature low-threshold avalanche effect in stepwise van-der-Waals homojunction photodiodes

Hailu Wang[1,2#], Hui Xia[1,2#], Yaqian Liu[1], Yue Chen[1,2], Runzhang Xie[1,2], Zhen Wang[1,2], Peng Wang[1,2], Jinshui Miao[1,2], Fang Wang[1,2], Tianxin Li[1,2], Lan Fu[3], Piotr Martyniuk[4], Jianbin Xu[5], Weida Hu[1,2*], Wei Lu[1,2,6*]*

[1]State Key Laboratory of Infrared Physics, Shanghai Institute of Technical Physics, Chinese Academy of Sciences, Shanghai 200083, China.
[2]University of Chinese Academy of Sciences, Beijing 100049, China.
[3]Department of Electronic Materials Engineering, Research School of Physics and Engineering, The Australian National University, Canberra, ACT 2601, Australia
[4]Institute of Applied Physics, Military University of Technology, 2 Kaliskiego St., 00-908 Warsaw, Poland
[5]Department of Electronic Engineering and Materials Science and Technology Research Center, The Chinese University of Hong Kong, Hong Kong SAR, China
[6]School of Physical Science and Technology, ShanghaiTech University, Shanghai 201210, China

#H. Wang and H. Xia contributed equally to this work.
*Corresponding author: huix@mail.sitp.ac.cn; (H. Xia); wdhu@mail.sitp.ac.cn (W. Hu); luwei@mail.sitp.ac.cn. (W. Lu)

Contents

Supplementary Note 1: Stepwise WSe_2 diodes prepared by selected-area-dry-etching 3

Supplementary Note 2: Statistical analysis on the breakdown voltage of WSe_2 diodes 5

Supplementary Note 3: The energy band structure of the stepwise layer junction 12

Supplementary Note 4: Comparison between InGaAs and stepwise WSe_2 diodes 13

Supplementary Note 5: Comparison of performance metrics for different types of APDs 18

Supplementary Note 6: Breaking the trade-off between photogain and signal-noise ratio 20

Supplementary Note 7: WSe_2 photodiode for large-dynamic-range imaging 22

Supplementary Note 8: WSe_2/hBN diodes 27

Supplementary References 31

Supplementary Note 1: Stepwise WSe_2 diodes prepared by selected-area-dry-etching

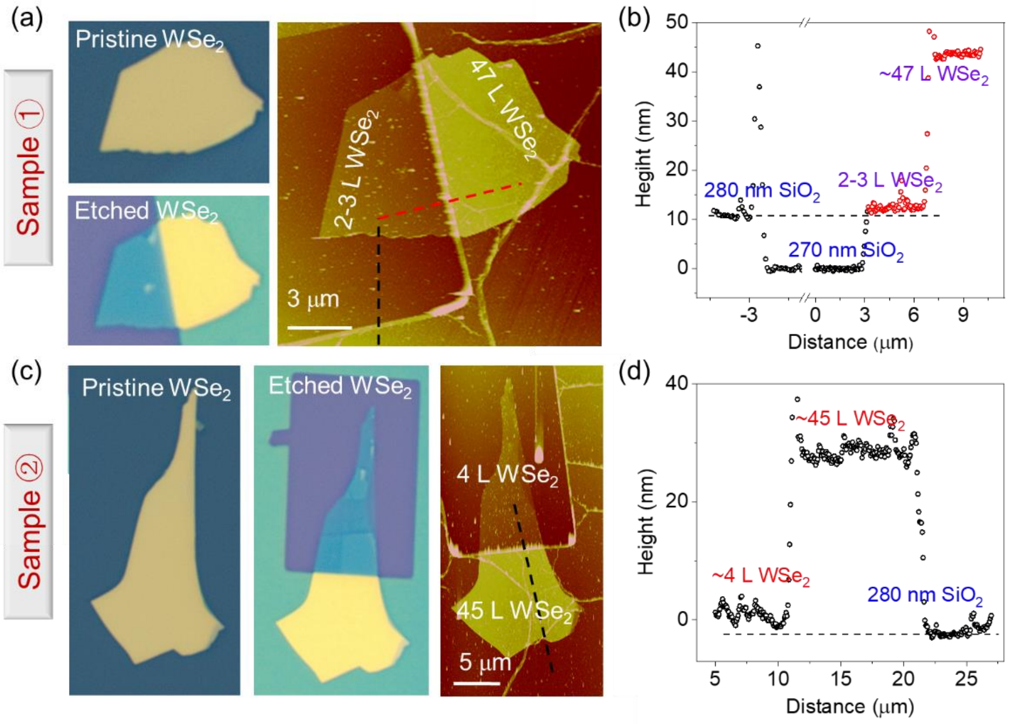

Supplementary Figure 1. Stepwise WSe_2 structure prepared by selected-area-dry-etching. Optical microscopy and atomic force microscopy (AFM) images of the pristine and etched WSe_2 for (a) Sample 1 and (c) Sample 2. Height profiles along the dashed lines extracted from AFM results for (b) Sample 1 and (d) Sample 2.

The stepwise vdW structure could be prepared by either mechanical exfoliation or a dry etching process. Here we show the latter approach and its outcome. First, a standard electron beam lithography process was utilized to expose a half section of the WSe_2 flake. After that, the reactive ion etching (RIE) technology is used to thin the exposed WSe_2 flake to monolayer thick. The etch depth can be precisely controlled by modulating plasma density (species include Ar and CF_4), pressure, temperature, and etch time. As shown in **Supplementary Figure 1**, half of the WSe_2 flakes are thinned from ~47/45 L to 4/2-3 L for Samples ① and ② respectively.

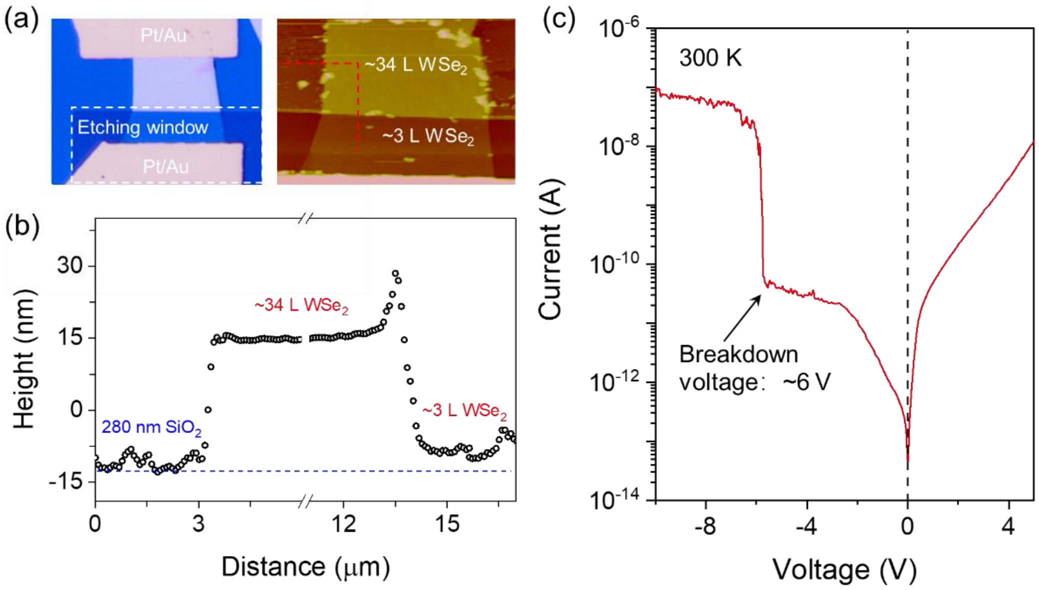

Supplementary Figure 2. Carrier multiplication characteristic of the WSe_2 diode fabricated by RIE etching. (a) Optical microscope and AFM image of the stepwise WSe_2 diode by RIE method. (b) Height profile showing the thickness of few-layer and multilayer WSe_2. (c) Dark *I*-*V* curve of the WSe_2 diode at 300 K.

Supplementary Figure 2a shows the optical microscope and atomic force microscope images of the stepwise WSe_2 diode fabricated in this way. For details, the multi-layer segment is 34 L in thickness, while the other part is thinned to 3 L. Pt/Au electrodes were deposited on both sides. **Supplementary Figure 2c** shows the *I*-*V* curve of the as-prepared WSe_2 diode in the dark at room temperature. One can find that the breakdown voltage is about -6 V, much higher than the devices fabricated by the mechanical exfoliation method. This could be caused by the impurities and defects in the RIE process, which will bring a high scattering effect and limit the performance.

Supplementary Note 2: Statistical analysis on the breakdown voltage of WSe_2 diodes

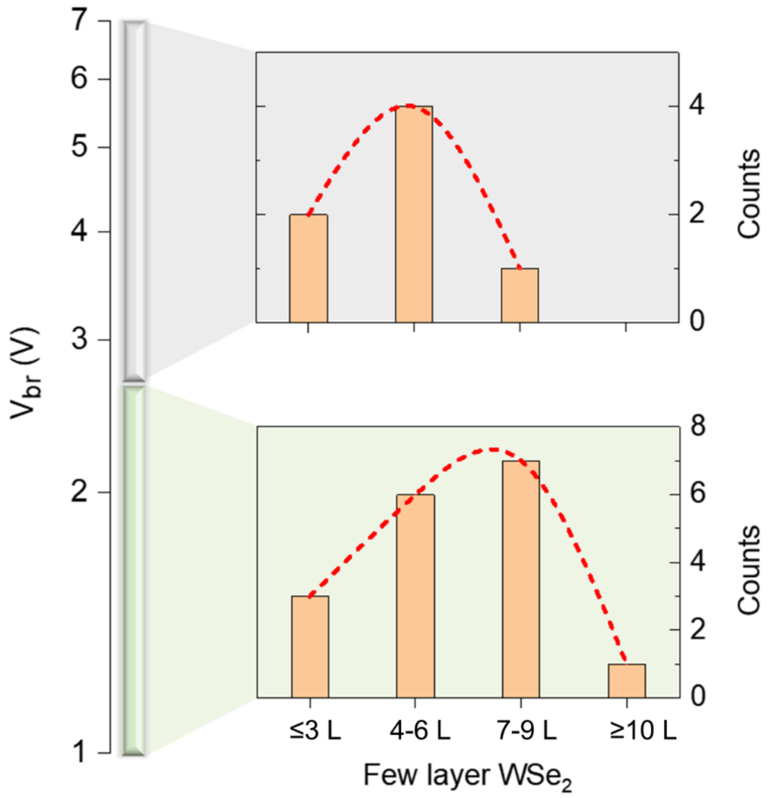

Supplementary Figure 3. Statistical analysis on the dependence of breakdown voltage on the few-layer WSe_2 thickness.

Considering that the depletion region primarily occurs in the few-layer WSe_2 (Figure 5b in the main text), charge carrier acceleration and impact ionization are likely to occur there. Therefore, our focus is on the impact of the few-layer thickness on the breakdown voltage. As shown in **Supplementary Figure 3**, we divided 25 devices into two groups: 1 V < $|V_{br}|$ <2.5 V and 2.5 V < $|V_{br}|$ < 7 V. Within each group, the devices are further subdivided into 4 sections based on the few-layer thickness: ≤3 L, 4-6 L, 7-9 L, and ≥10 L. This approach will enable us to easily establish the relationship between the breakdown voltage and the thickness of WSe_2. As shown in **Supplementary Figure 3**, high breakdown voltage (2.5-7 V) is frequently observed in thin devices, with the thickness of the few-layer WSe_2 being approximately ~4 L. In contrast, a low breakdown voltage (1-2.5 V) is closely associated with thick devices, where the thickness of the few-layer WSe_2 is approximately ~7 L. This is reasonable because the bandgap is inversely proportional to the thickness of TMD materials. Therefore, for

avalanche breakdown, thinner/thicker materials (with larger/smaller bandgaps) will require a higher/lower external bias voltage. Detailed information on thickness combinations and breakdown voltage for 25 WSe_2 devices is listed in **Supplementary Table 1**.

Based on the statistical analysis results, it is evident that the selection of thickness combinations plays a pivotal role in determining the avalanche breakdown voltage. It is essential to emphasize that the avalanche process is influenced by multiple intrinsic factors such as doping concentration, the width of the avalanche region, bandgap, and even the device shape (especially for low-dimensional materials). Therefore, it is crucial to recognize that while thickness combinations are significant, they are not the sole determinant in achieving low-threshold avalanche devices.

Supplementary Table 1. Summary of the stepwise WSe_2 diodes with different thickness combinations.

Device Number (#)	Few-layer WSe_2 (layer)	Multilayer WSe_2 (layer)	Breakdown voltage (V)
1	~6	~29	-1.4
2	~5	~36	-2.1
3	~3	~17	-1.9
4	~6	~28	-1.4
5	~3	~27	-1.6
6	~8	~25	-1.7
7	~7	~33	-1.4
8	~5	~75	-1.9
9	~7	~19	-1.5
10	~3	~55	-1.4
11	~8	~29	-1.5
12	~7	~22	-1.8
13	~7	~38	-1.5
14	~6	~53	-1.4
15	~7	~45	-1.6
16	~5	~27	-1.5
17	~13	~22	-3.0
18	~10	~53	-2.2
19	~3	~13	-5.0
20	~5	~54	-5.4
21	~4	~46	-5.0
22	~7	~36	-4.8
23	~4	~45	-3.5
24	~3	~16	-4.3
25	~6	~40	-3.0

a) The thickness of the monolayer WSe_2 is about 0.7 nm.

More experimental data of twenty-five WSe_2 diodes, including optical microscope images, AFM images, height profiles extracted from AFM results, and dark *I*-*V* curves, are shown in **Supplementary Figures 4-10**.

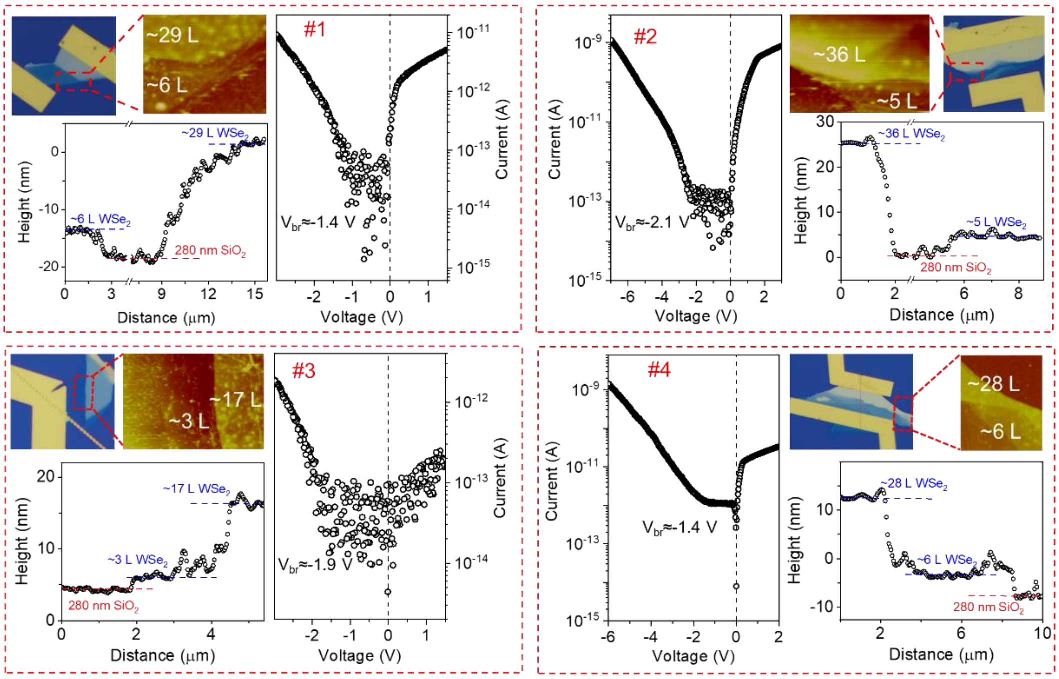

Supplementary Figure 4. Optical microscope images, AFM images, height profiles extracted from AFM results, and dark *I*-*V* curves of Numbers #1, #2, #3, and #4 stepwise WSe_2 diodes.

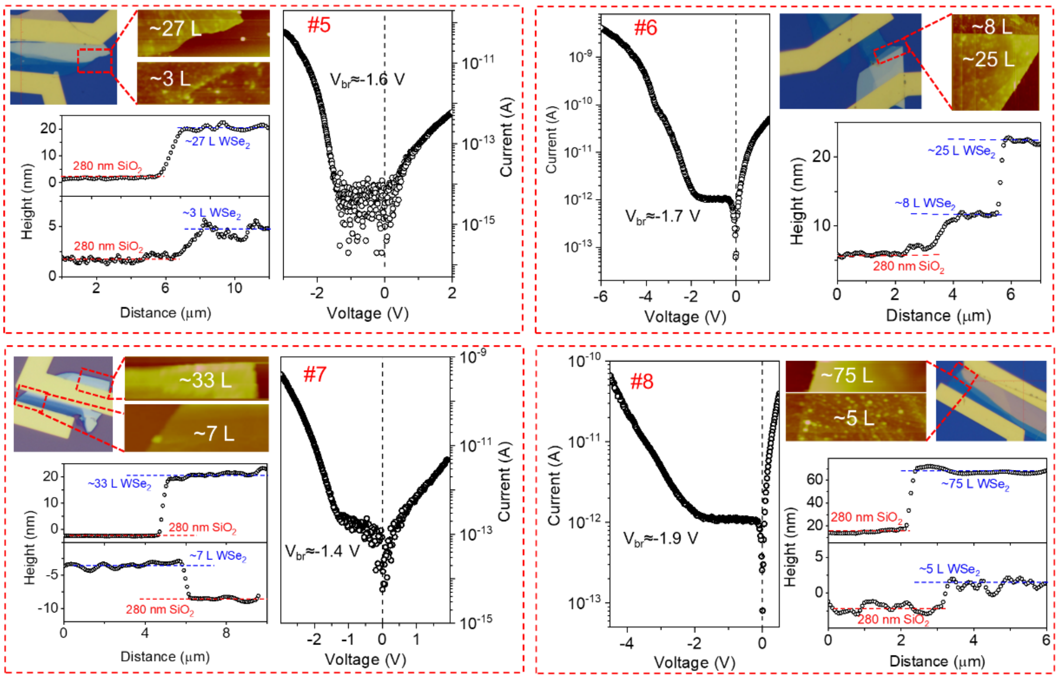

Supplementary Figure 5. Optical microscope images, AFM images, height profiles

extracted from AFM results, and dark *I-V* curves of Numbers #5, #6, #7, and #8 stepwise WSe_2 diodes.

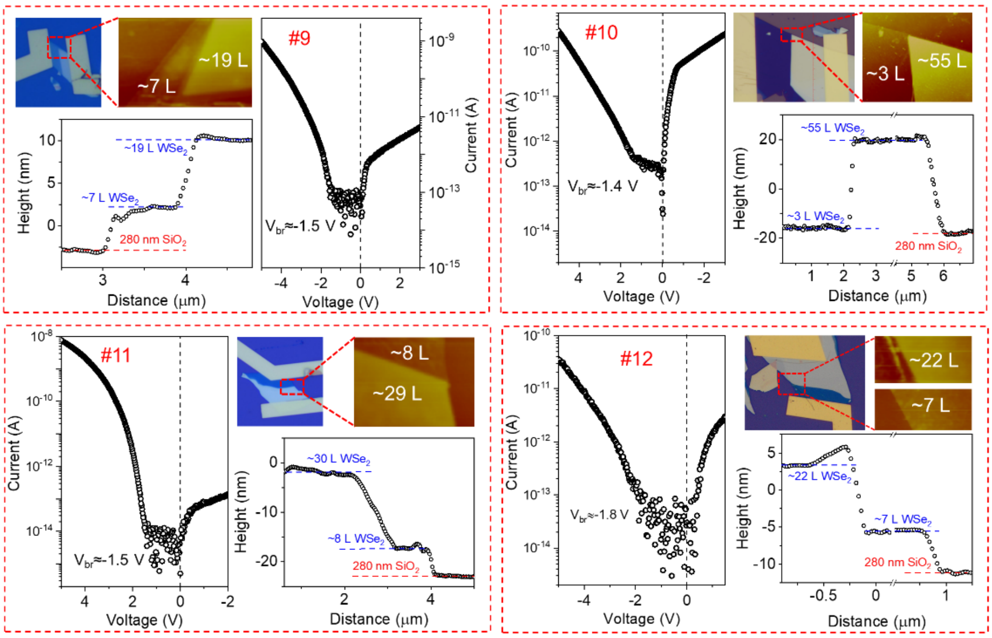

Supplementary Figure 6. Optical microscope images, AFM images, height profiles extracted from AFM results, and dark *I-V* curves of Numbers #9, #10, #11, and #12 stepwise WSe_2 diodes.

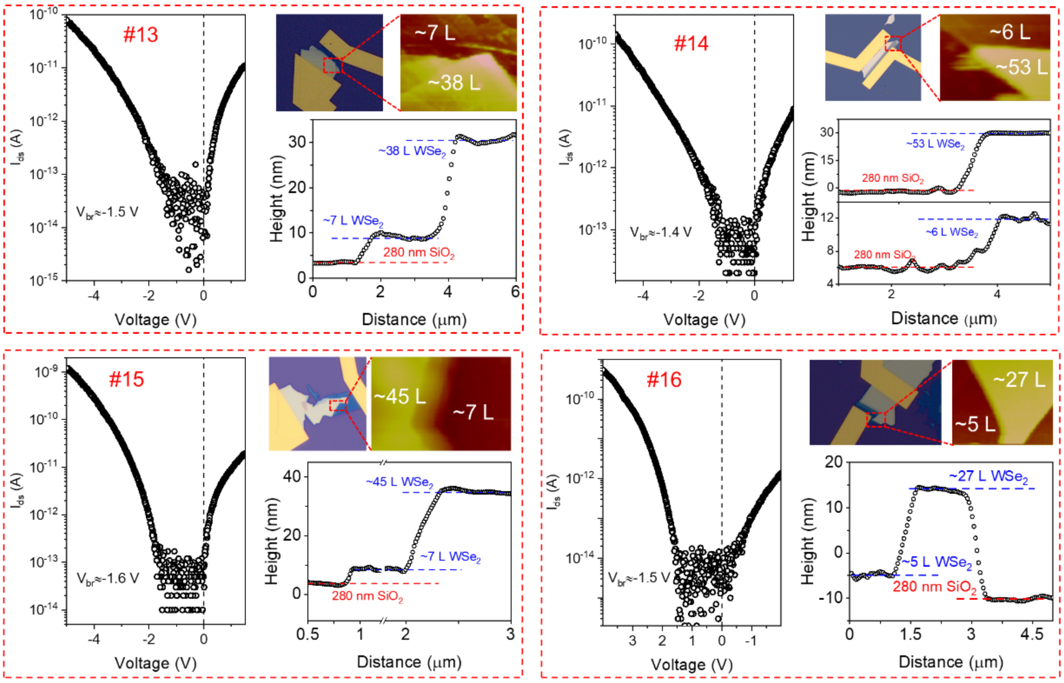

Supplementary Figure 7. Optical microscope images, AFM images, height profiles extracted from AFM results, and dark *I-V* curves of Numbers #13, #14, #15, and #16 stepwise WSe_2 diodes.

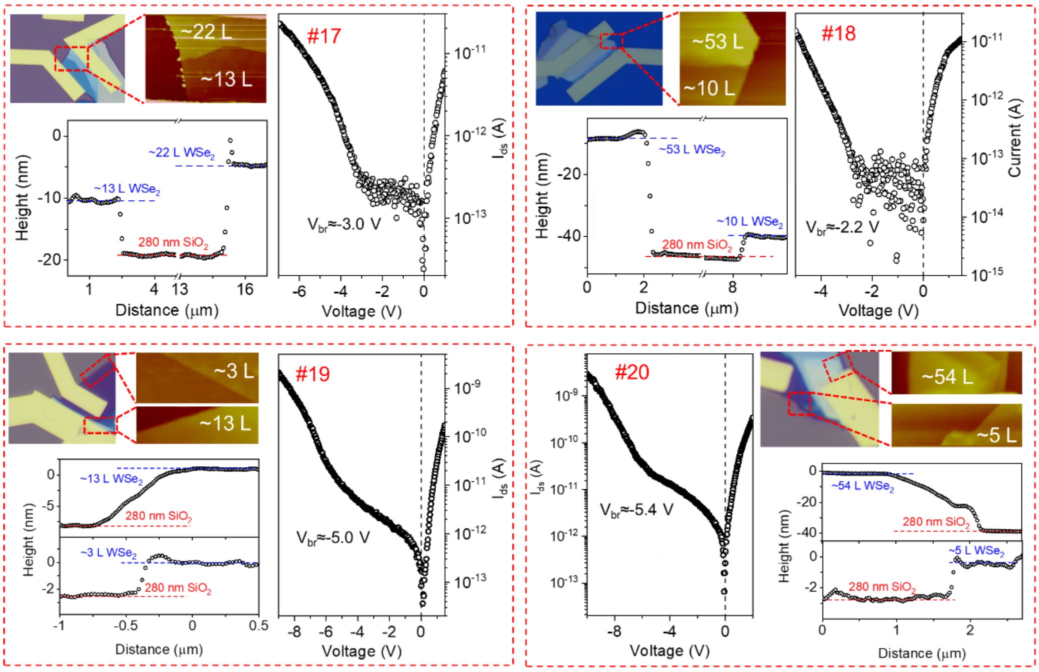

Supplementary Figure 8. Optical microscope images, AFM images, height profiles extracted from AFM results, and dark *I-V* curves of Numbers #17, #18, #19, and #20 stepwise WSe_2 diodes.

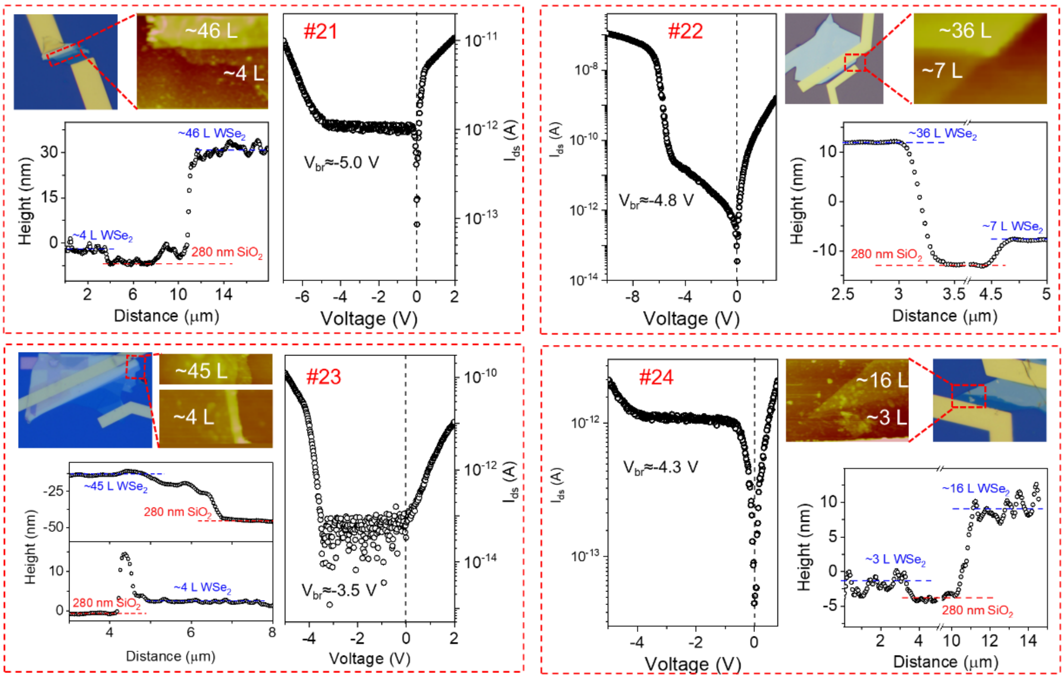

Supplementary Figure 9. Optical microscope images, AFM images, height profiles extracted from AFM results, and dark *I-V* curves of Numbers #21, #22, #23, and #24 stepwise WSe_2 diodes.

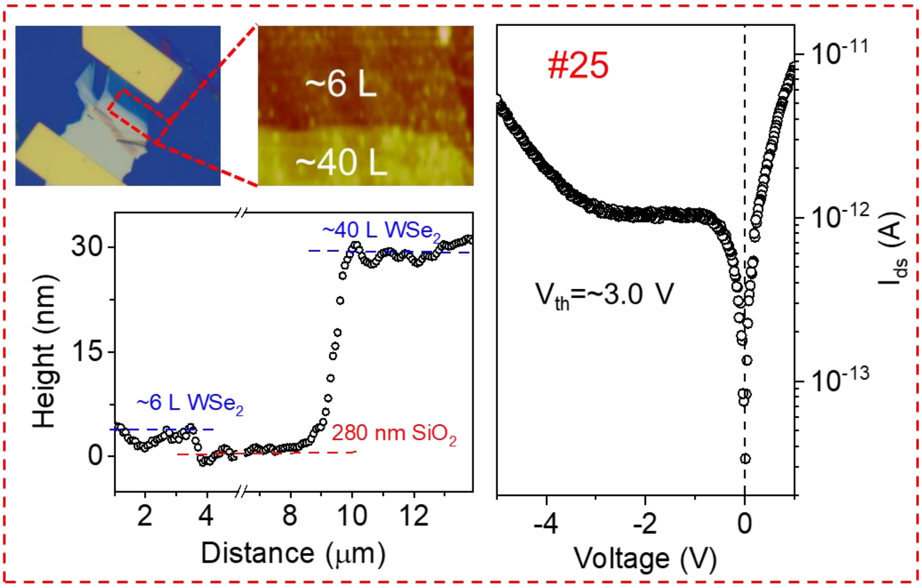

Supplementary Figure 10. Optical microscope images, AFM images, height profiles extracted from AFM results, and dark *I-V* curves of Numbers #25 stepwise WSe_2 diodes.

Supplementary Note 3: The energy band structure of the stepwise layer junction

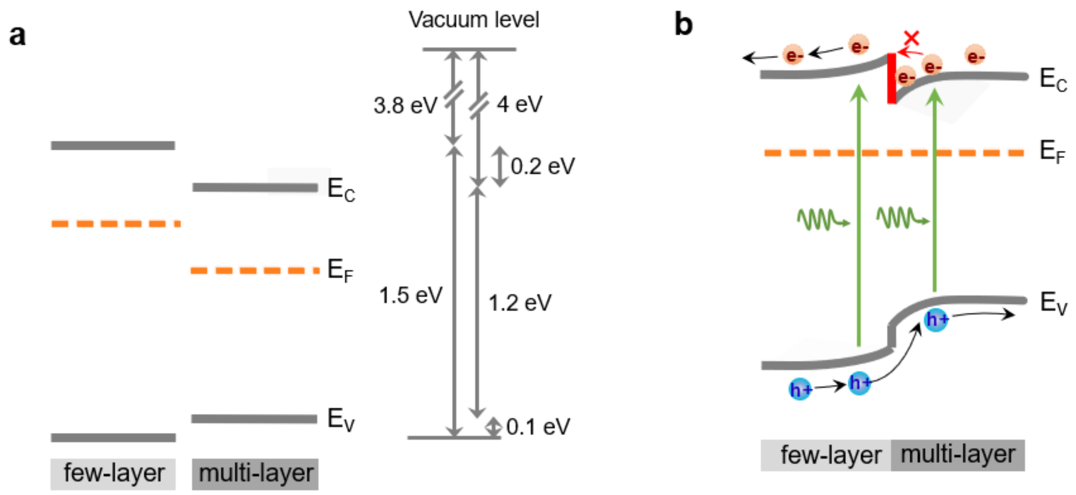

Supplementary Figure 11. Energy band alignment and charge carrier transport in the stepwise layer junction. (a) Band alignment of few- and multi-layer WSe_2 before contact. (b) Energy band diagram and the photocarrier transport process.

The electron Fermi-level of the n^--WSe_2 film shifts downward as the layer thickness continuously increases. It indicates that the energy band is aligned as a type I isotype heterojunction (**Supplementary Figure 11a**). **Supplementary Figure 11b** illustrates the band-bending and photocarrier transport behaviors of the stepwise layer junction. The multi-/few- layer is bent downward/upward significantly. It gives rise to a potential barrier in the conduction band, which denies the electron transport from multi-layer to few-layer direction. For this reason, the multi-layer segment will contribute little to the photoresponse of the device.

Those characters help to understand the rectifying characteristic of the homojunction. when the device is positively biased (a positive bias is applied to the multilayer part), the conduction band of the few-layer segment will be lifted, and the electron barrier will be lowered. It thus leads to a forward conducting behavior. In the negatively biased case, the barrier will be amplified, thus resulting in a cut-off behavior.

Supplementary Note 4: Comparison between InGaAs and stepwise WSe_2 diodes

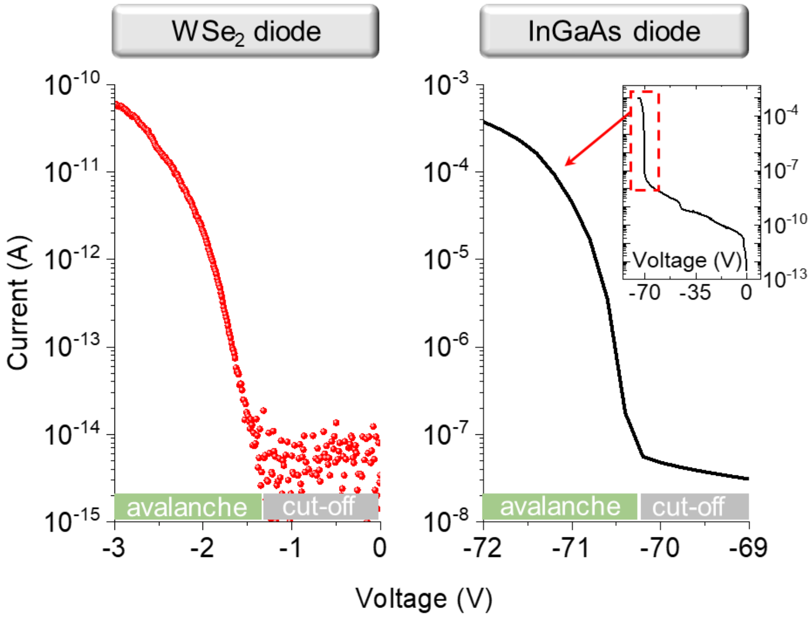

Supplementary Figure 12. Comparison of *I*-*V* curves between WSe_2 and InGaAs avalanche diodes in the dark. The inset: full-scale *I*-*V* curves of InGaAs avalanche diodes.

We compare the *I*-*V* curves of the WSe_2 device with those of commercial InGaAs avalanche diodes. As shown in **Supplementary Figure 12**, both kinds of devices experience a ~10^4 times increase of current after avalanching. And, more importantly, their current climbs almost at the same rate, dV/d(lgI)≈400 mV/dec.

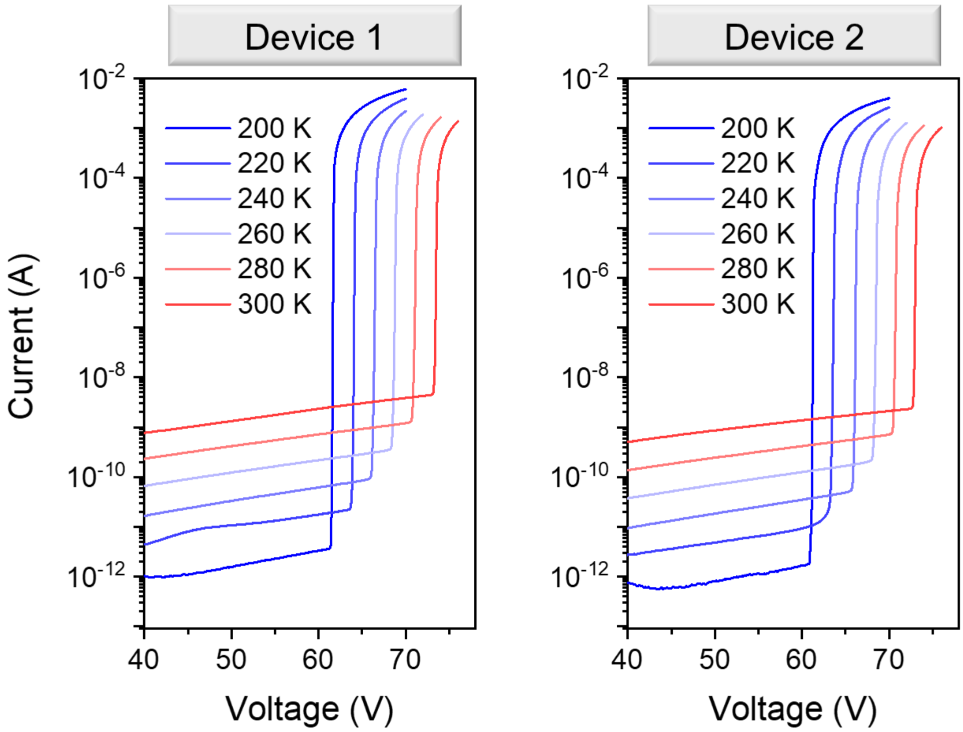

Supplementary Figure 13. Variable temperature *I-V* curves of two InGaAs avalanche diodes.

Supplementary Figure 13 shows the variable temperature *I-V* curves of two commercial InGaAs avalanche diodes. It is featured by a high breakdown voltage, up to ~73 V at room temperature, and a positive temperature coefficient, in which the breakdown voltage decreases with a decreasing temperature.

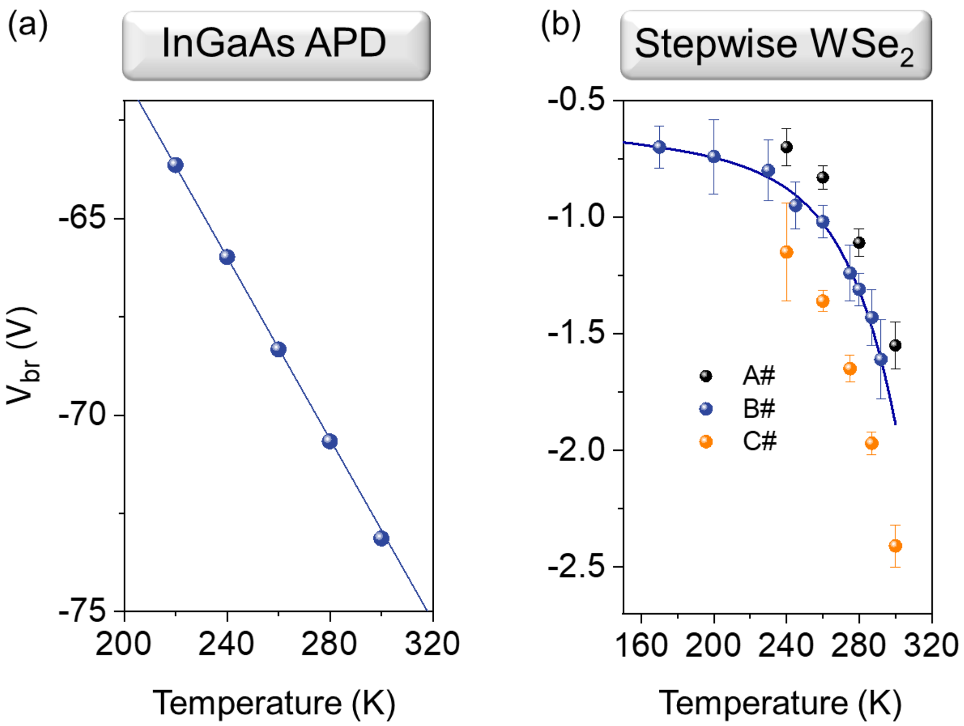

Supplementary Figure 14. Dependence of breakdown voltage on the operating temperature in (a) InGaAs and (b) stepwise WSe_2 avalanche diodes. In panel (a), the scatter line represents the experimental data, while the solid line is included for visual guidance. The scatter lines in panel (b) are the experimental results, while the solid line is the fitting curve. A#, B#, and C# represent three different stepwise WSe_2 diodes.

Supplementary Figure 14 shows the dependence of breakdown voltage on the operating temperature or InGaAs and WSe_2 avalanche diodes. For InGaAs, the breakdown voltage transits from 73.14 V@300 K to 63.65 V@220 K. The temperature coefficient is then determined as positive. For $WeSe_2$ diodes, taking the B# device as an example, the breakdown voltage transits from ~-1.61 V @ 292 K to ~-0.7 V @ 170 K. The temperature coefficient is also determined as positive, verifying that the reverse breakdown comes from the carrier avalanche process. Of particular interest is the significant decrease in the temperature coefficient as the temperature falls below 250 K (**Supplementary Figure 14b**, blue line). This directly leads to a saturation tendency of the breakdown voltage. By fitting the curve with a double-exponential equation: V_{br}=-A×$e^{\frac{T-T_0}{\alpha}}$-B×$e^{\frac{T}{\beta}}$, we obtain a final limit value of -0.57 V (0.35E_g/q). Such a characteristic not only justifies our claim of the threshold-limit-avalanche but also explains why the breakdown voltage could be lower than E_g/q. This is because in the limit case, the internal built-in voltage also contributes to the avalanche process, thereby lowering the standard for external bias voltage, for example, 0.44E_g/q.

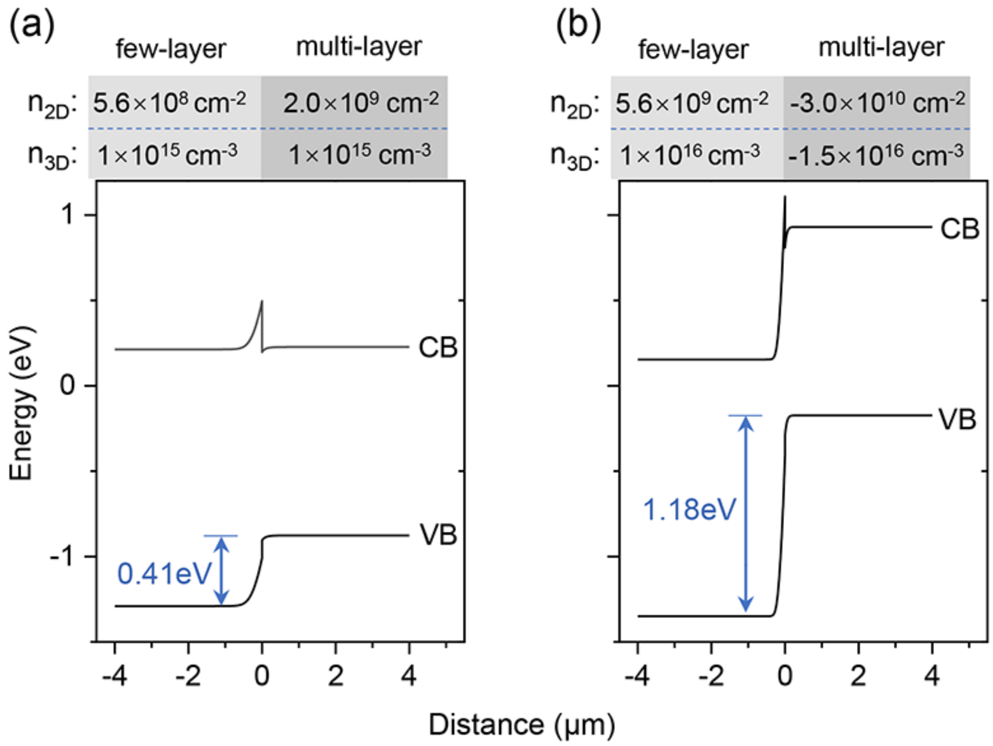

Supplementary Figure 15. Calculated energy-band structure of WSe_2 diode at zero bias voltage. (a) without and (b) with considering the discrepancy of doping characteristics between the few- and multi-layer segments. n_{2D} and n_{3D} represent the two- and three-dimensional carrier concentration, that are counted in the form of cm^{-2} and cm^{-3}.

For diode at avalanching, there are two voltage components contributing to the electron acceleration and subsequent impact ionization, external bias voltage and internal built-in potential. Generally, the external bias voltage is up to $100E_g/q$ in Si and GaN avalanche devices, while the built-in potential is at a low level, ~$0.7E_g/q$. This makes people easily neglect the contribution of the latter.

In a low threshold avalanche device, however, the breakdown voltage is lowered to ~E_g/q. In this regard, the contribution of built-in potential to the avalanche performance should not be neglected. To clarify this kind of issue, we further perform a numerical simulation. The theoretical model is established with a commercial software package (Sentaurus-TCAD). It considers the stepwise geometry of the WSe_2 device, where the two distinct segments are set as 5.6 (8 L) and 20.3 nm (29 L) in thickness. The electron affinity and bandgaps are 3.7 and 1.6 eV, 4.0 and 1.2 eV, for few- and multi-layer parts,

respectively [1].

As shown in **Supplementary Figure 15** and **Supplementary Table 2**, the built-in potential arises from two aspects: the band-offset and the Fermi-level drop (induced by different doping polarities and concentrations in the counterpart segments). According to the simulation, the band offset solely leads to an internal potential of 0.41 V (close to $\frac{1.6\ \text{eV}-1.2\ \text{eV}}{q}$=0.4 V, **Supplementary Figure 15a**), while the latter one rises to 1.18 V and is even higher (**Supplementary Figure 15b**). If we assume that there is no energy loss during the electron acceleration process, the minimum voltage required for the avalanche would be lowered to $\frac{E_g=1.6\ \text{eV}}{q}$-1.2 V (1.52 V)=0.4 V (0.08 V) (**Supplementary Table 2**). This explains why the breakdown voltage could further decrease as temperature drops.

Supplementary Table 2. Summary on the built-in potential of $WeSe_2$ diode and minimum bias-voltage required for avalanche at low temperatures.

Minimum energy (required for avalanche)	Built-in potential		Minimum bias-voltage (required for avalanche) at low temperatures
	Band-offset	Fermi level drop	
E_g, ~1.6 eV	~0.4 V	0.5 E_g/q	0.4 V
		0.7 E_g/q	0.08 V

Supplementary Note 5: Comparison of performance metrics for different types of APDs

Supplementary Table 3. Comparison of performance metrics for different types of APDs.

APD structure		Operating temperature	Temperature coefficient	Breakdown voltage	Gain	Dark current	Ref.
Bulk material	Si PIN junction	300 K	Positive	-150 V	~100 @-150 V	~50 pA	2
	InGaAs PIN junction	300 K	Positive	-55 V	~30 @-55 V	~40 nA	3
	Si-Ge PIN junction	300 K	Positive	-25 V	~30 @-25 V	~100 pA	4
	GaN PIN junction	300 K	Positive	-92 V	~300 @-92 V	~100 pA	5
	Ge PIN junction	300 K	Positive	-30 V	~100 @-30 V	~300 nA	6
	AlGaN Schottky junction	/	/	-50 V	1560 @-68 V	~10 pA	7
Two-dimensional material	WSe_2/WS_2 PN heterojunction	/	/	-8.5 V	~300 @-16.5 V	~100 pA	8
	MoS_2/WSe_2 PN heterojunction	300 K	/	-6.5 V	~5 @-10 V	~100 pA	9
	MoS_2 PN homojunction	100 K	/	-4.5 V	~100 @-10 V	~1 pA	10
	MoS_2 Uniform n-doping	300 K	positive	-40 V	/	~1 μA	11
	BP/InSe PN heterojunction	80 K	Negative	-4.8 V	~100 @-2 V	~100 pA	12
	**WSe_2 Stepwise homojunction**	**300 K**	**Positive**	**-1.6** V	**~200 @-2 V**	**~10 fA**	**This work**

We have summarized the performance metrics of several typical avalanche photodetectors (APDs) based on bulk materials and two-dimensional layered materials, as shown in **Supplementary Table 3**. One can find that APDs based on conventional bulk materials show good performance at room temperature, but often require high breakdown voltage (more than 25 V). Besides, all bulk materials-based APDs show a positive temperature coefficient, which is a typical feature of the avalanche multiplication process. In comparison, APDs based on two-dimensional materials have relatively low breakdown voltage due to their small size but often operate at low-temperature conditions. Our work proposes a WSe_2 stepwise homojunction diode with a low avalanche threshold voltage of about 1.6 V, a low dark current of about 10 fA, and a large avalanche gain of about 200 at room temperature. Importantly, the proposed WSe_2 diodes demonstrate a positive temperature coefficient, the same as the traditional bulk APDs.

Supplementary Note 6: Breaking the trade-off between photogain and signal-noise ratio

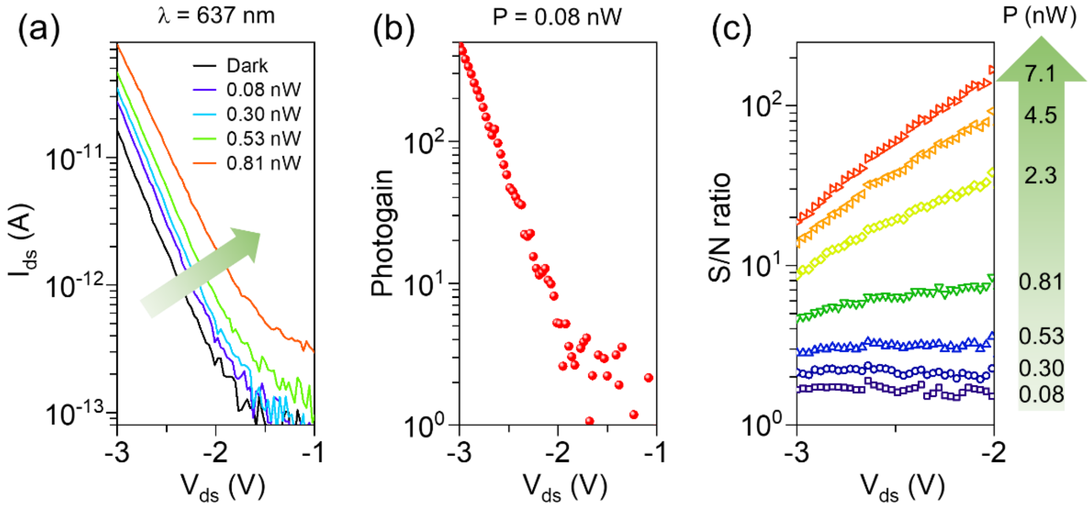

Supplementary Figure 16. Photo-gain and signal-noise (S/N) ratio of the stepwise WSe_2 diode. (a) Dark and photo-excited *I-V* curves of the device after avalanching. (b) Photogain of the device under an illumination intensity of 0.08 nW. (c) Signal-noise ratio of the device while it is operated at Geiger mode. The intensity of the excitation light source ranges from 0.08 to 7.1 nW.

In a traditional avalanche diode, the dark current rapidly catches up with the photocurrent after avalanching. It leads to difficulty in resolving signals from background noise in Geiger mode. Here we show the advantage of the stepwise WSe_2 diode in avalanching applications, in which the trade-off between gain and signal-noise-ratio is broken. As depicted in **Supplementary Figure 16a**, the *I-V* curves under illumination shift upward as compared with those in the dark. It results in a high photogain, up to 470 (**Supplementary Figure 16b**). At the same time, the ratio of photocurrent to dark-current (signal-to-noise ratio) reaches an unprecedented level, up to 167 (**Supplementary Figure 16c**). Those characters allow the device to conveniently work at Geiger mode, and detect light signals down to femtowatt level.

Experimentally, the lowest illumination intensity that the device can respond to is ~24 fW. It is calibrated by the equation, $I = \frac{P}{A_{spot}} \times A_{device}$, where I is the

illumination intensity normalized to the device, P is the laser power (0.08 nW), A_{spot} is the area of the laser spot (0.785 mm^2, 1mm in spot diameter), A_{device} is the device area (~230 μm^2). Considering that the laser wavelength is 637 nm, the lowest phonon number that the device can detect is approximately 7.7×10^4.

Supplementary Note 7: WSe_2 photodiode for large-dynamic-range imaging

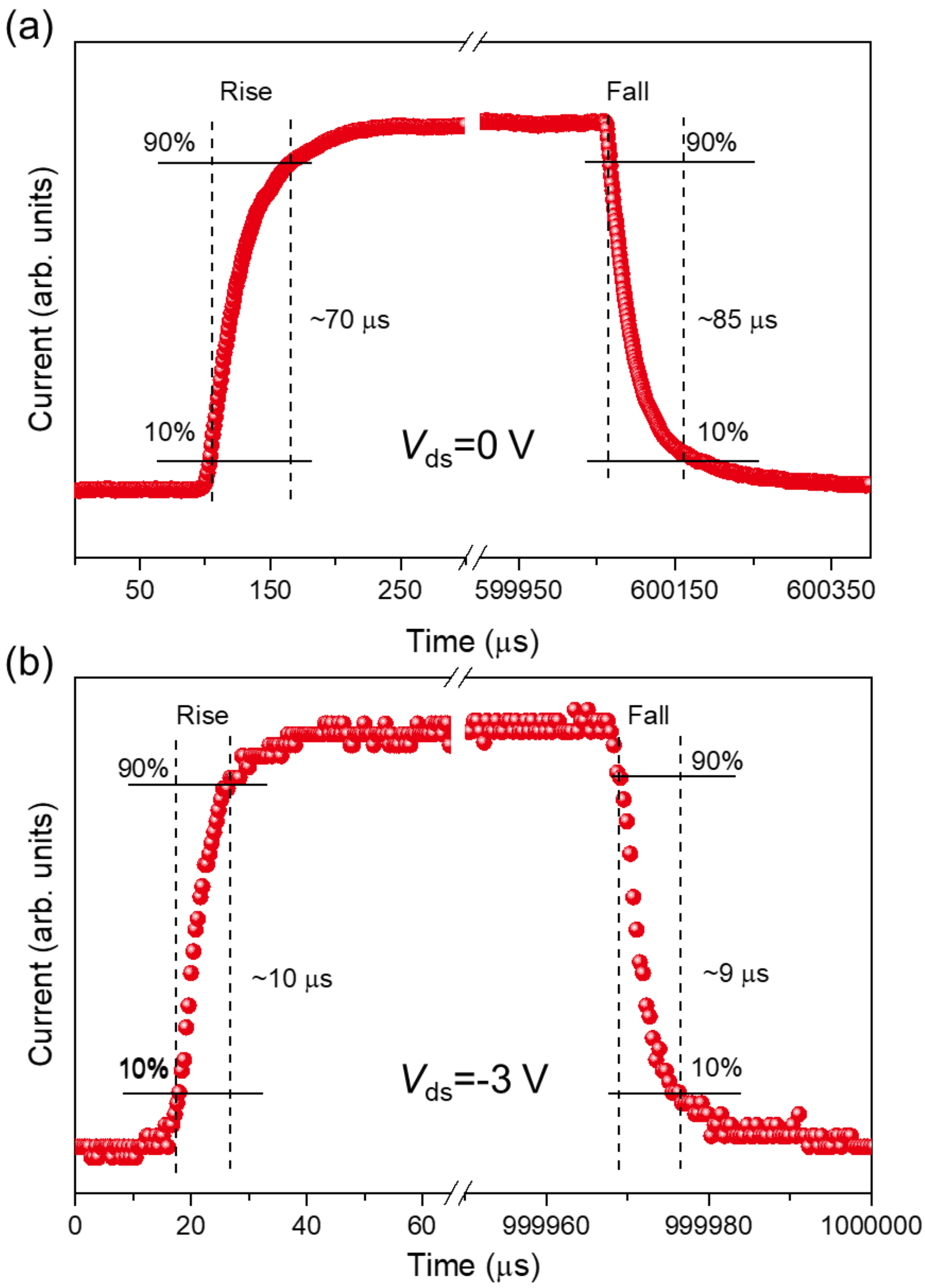

Supplementary Figure 17. Time-resolved photoresponse of the device under 520 nm laser illumination at (a) V_{ds} = 0 V and (b) -3 V.

Response time is an important performance parameter for a photodetector in practical applications. The rising/falling time is defined as the total time needed for the photocurrent to rise (fall) from 10% (90%) to 90% (10%) of the peak. **Supplementary Figure 17** shows the rising/falling time of the proposed WSe_2 diode at 0 and -3 V. One can find that the rising/falling increased from 70/85 μs to 10/9 μs with the growing bias

voltages. Worth noting that the response time could be limited by the RC time constant of the measurement system.

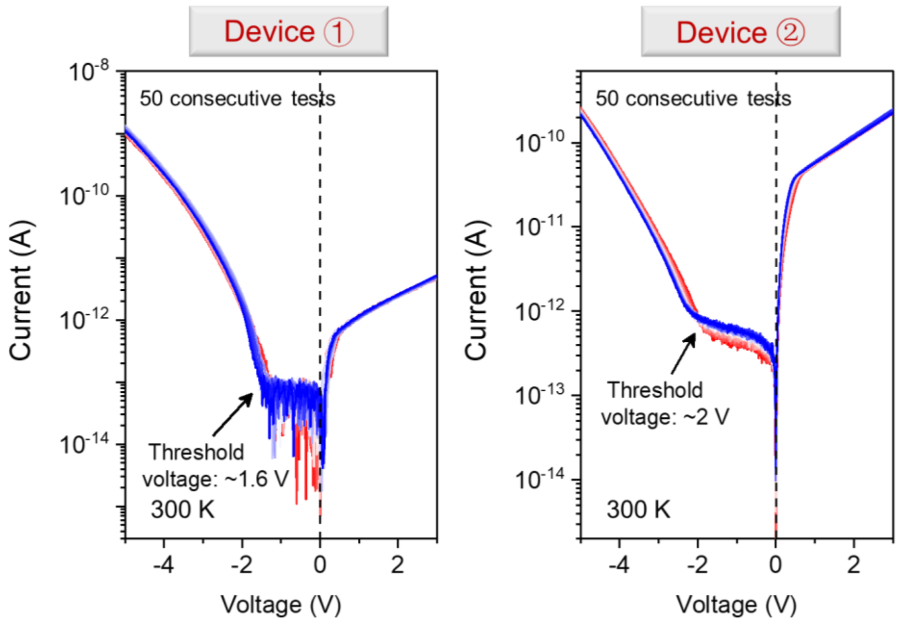

Supplementary Figure 18. Dark *I-V* curves of 50 consecutive tests for WSe_2 diodes (a) Device ① and (b) ②.

Stability and repeatability are also very important for the practical application of the photoelectric device. We have demonstrated the stability testing of the proposed WSe_2 diodes to confirm the device degradation after measurement and overtime at room temperature, as shown in **Supplementary Figure 18**. One can find that the breakdown voltages are almost unchanged after 50 consecutive tests, remaining at 1.6 and 2 V for Device ① and ②.

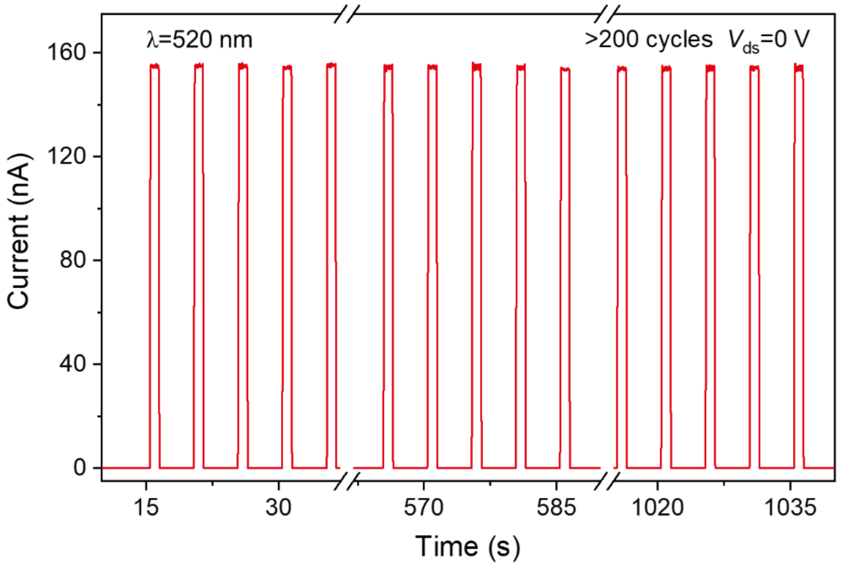

Supplementary Figure 19. Photoswitching response of the WSe_2 diode under the illumination of 520 nm at V_{ds}=0 V.

To verify the stability of the WSe_2 diodes under laser illumination, we also measure the photoswitching response at zero bias under a periodical on/off 520 nm laser illumination at room temperature, as shown in **Supplementary Figure 19**. The current rapidly increases when the laser is on and recovers to the dark state quickly when it is off. No obvious baseline drift in time-resolved photocurrent is observed with >200 cycles of modulated laser illumination, which confirms the good photoswitching stability of the devices.

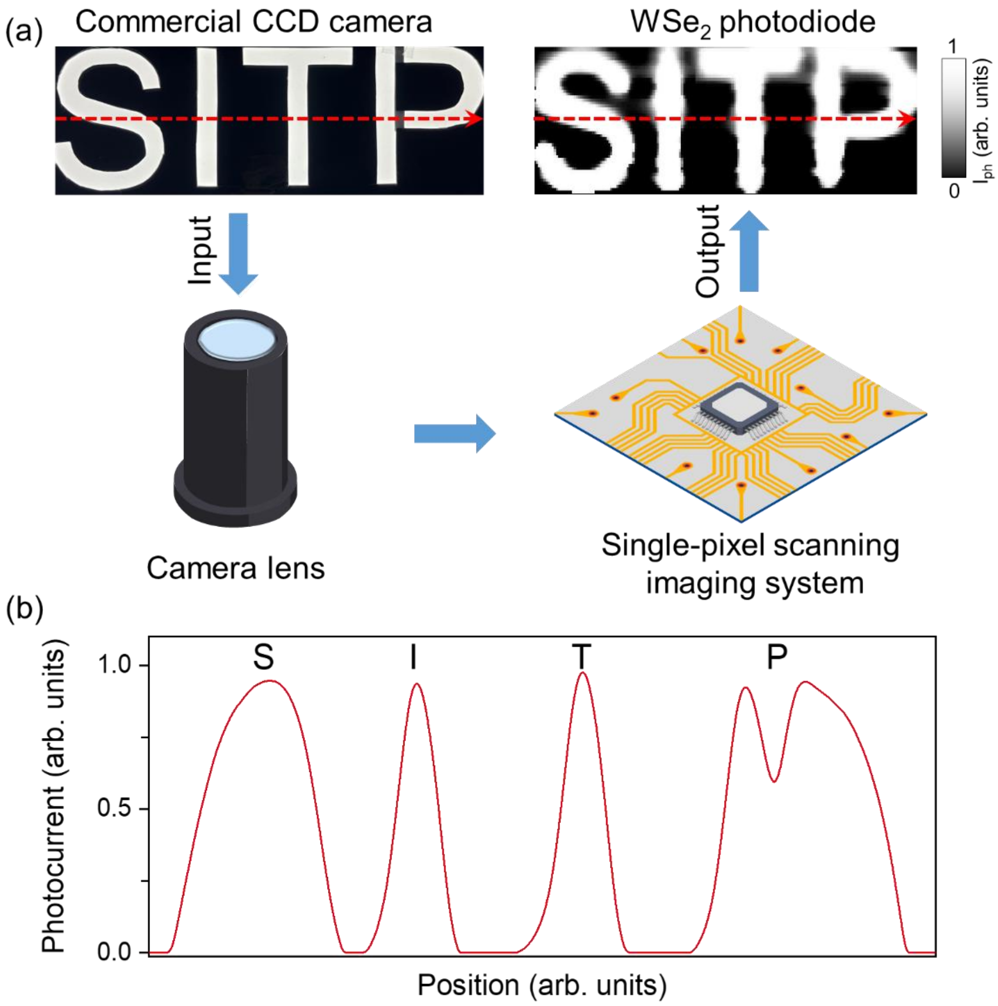

Supplementary Figure 20. Imaging application of the WSe_2 photodiode. (a) Schematic diagram of the single-pixel scanning imaging system. SITP (the abbreviation of Shanghai Institute of Technology and Physics) letter graphics under white light illumination are used as imaging targets. (b) Normalized photocurrent profile along the horizontal direction of the target (marked by red dotted lines in imaging photographs).

To demonstrate the practical feasibility of the WSe_2 photodiode with high dynamic range and high sensitivity, we further performed the actual imaging measurements by a homemade imaging system at room temperature and ambient conditions. The main components of the imaging system are composed of a photodetector, an optical system (including lens, filter, and so on), a two-dimensional motor controller, and a current preamplifier. The prepared WSe_2 stepwise junction photodiode was integrated into the imaging system as the detector and operated at V_{ds}=0 V. As shown in **Supplementary Figure 20a**, a SITP hollow

graphic under white light illumination is used as the imaging target. The light of the target was focused on the detector through the optical system. The motor controller was used to drive the detector to scan the whole target in two-dimensional pixel by pixel. The generated photocurrent signals of each pixel were then amplified by the preamplifier and recorded to obtain a two-dimensional image. **Supplementary Figure 20a** (upper right panel) shows the imaging result of the SITP letters. One can find that a clear SITP pattern with sharp boundaries is obtained. We further extract the photocurrent profile along the horizontal direction of the target, shown in **Supplementary Figure 20b**. The photocurrent profile shows an obvious increase with the position movement from the background to the pattern body, which indicates the growing optical power from the background to the pattern edge and pattern body. The superior photoelectric performance of our WSe_2 photodiodes is confirmed by successfully identifying the difference between very high- and low-brightness targets and obtaining a clear imaging result.

Supplementary Note 8: WSe_2/hBN diodes

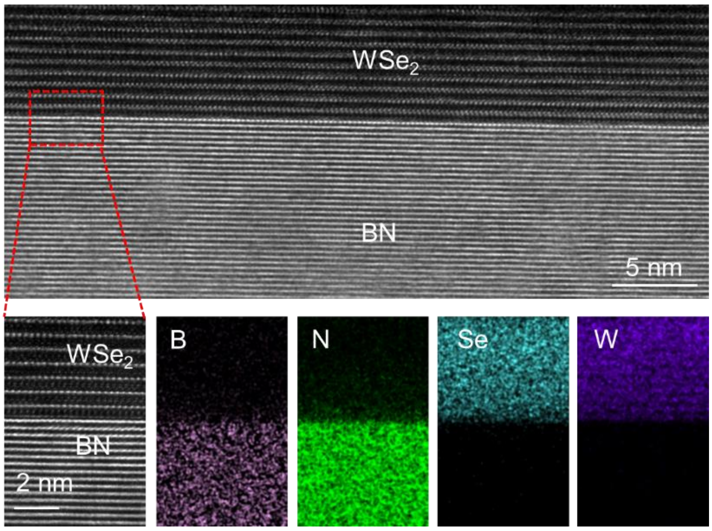

Supplementary Figure 21. High-resolution TEM images near the WSe_2/hBN interface.

We initially transferred the hBN layer onto the Si/SiO_2 substrate and then transferred WSe_2 onto the surface of hBN to create the WSe_2/hBN diodes. Additionally, we conducted high-resolution TEM tests to confirm the formation of a high-quality WSe_2/BN interface after the transfer processes. As illustrated in **Supplementary Figure 21**, the TEM images reveal no noticeable impurities or defects near the WSe_2/hBN interface, enabling us to investigate the impact of the hBN layer on device performance more effectively.

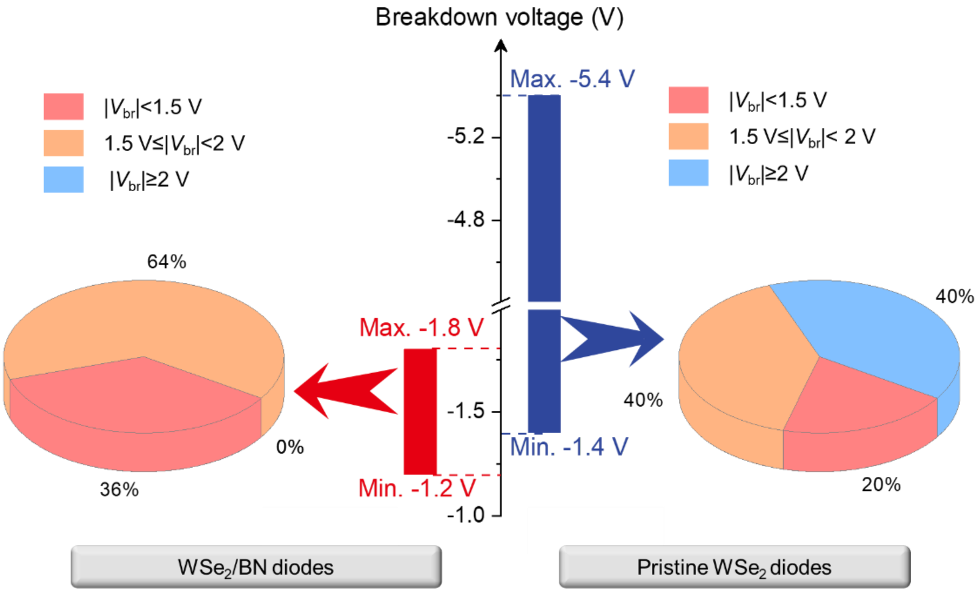

Supplementary Figure 22. Comparison of threshold voltage distribution for WSe_2/hBN (left panel) and pristine WSe_2 diodes (right panel). The red and blue bars indicate the threshold voltage distribution ranges of 11 WSe_2/hBN and 25 WSe_2 diodes, respectively.

We fabricated additional WSe_2/hBN diodes and conducted a statistical analysis of the threshold voltage data for 11 WSe_2/hBN diodes in comparison to 25 pristine WSe_2 diodes. The results are presented in **Supplementary Figure 22**. Among these devices, the threshold voltage of the WSe_2/hBN and pristine WSe_2 diodes range from -1.2 to -1.8 V (indicated by the red bars) and from -1.4 to -5.4 V (indicated by the blue bar), respectively. Clearly, WSe_2/hBN diodes exhibit lower minimum threshold voltages (-1.2 V, devices #1 and #2 in **Supplementary Figure 23**) and a more concentrated distribution compared to pristine WSe_2. For a more detailed analysis, we divided the threshold voltage into three ranges: $|V_{br}| < 1.5$ V, 1.5 V$\leq |V_{br}| <$2 V, and $|V_{br}|\geq$2 V. Among them, the proportions of WSe_2/hBN and pristine WSe_2 diodes in the three threshold voltage ranges are 36%, 64%, 0%, and 20%, 40%, 40%, respectively. This further indicates that the overall performance of WSe_2 diodes is indeed improved when a hBN layer is used as the substrate, due to the reduced scattering processes. The detailed experimental data, including photo-excited *I*-*V* curves and threshold voltage distribution for 11 typical WSe_2/hBN diodes, are shown in **Supplementary Figures 23-25**.

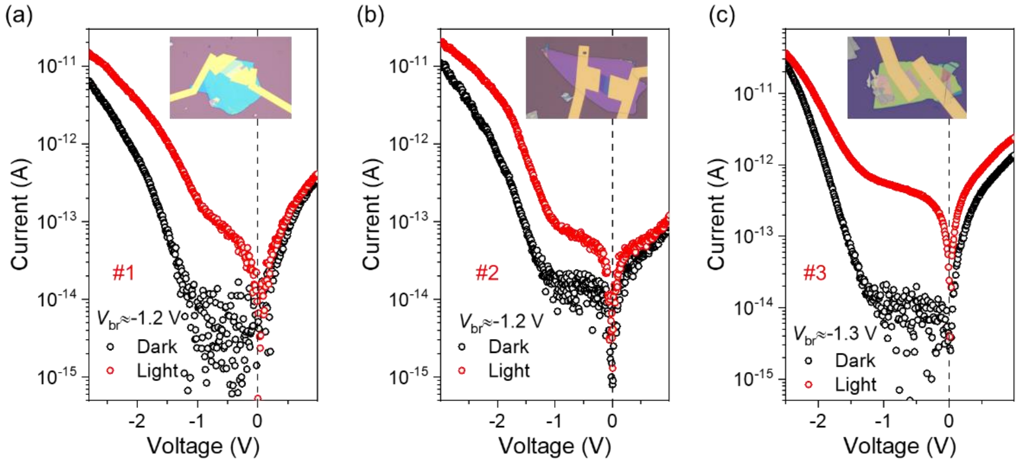

Supplementary Figure 23. *I-V* curves of Numbers (a) #1, (b) #2, and (c) #3 WSe_2 diodes encapsulated with BN in the dark and under illumination at 300 K. Inset: optical microscope images of the devices.

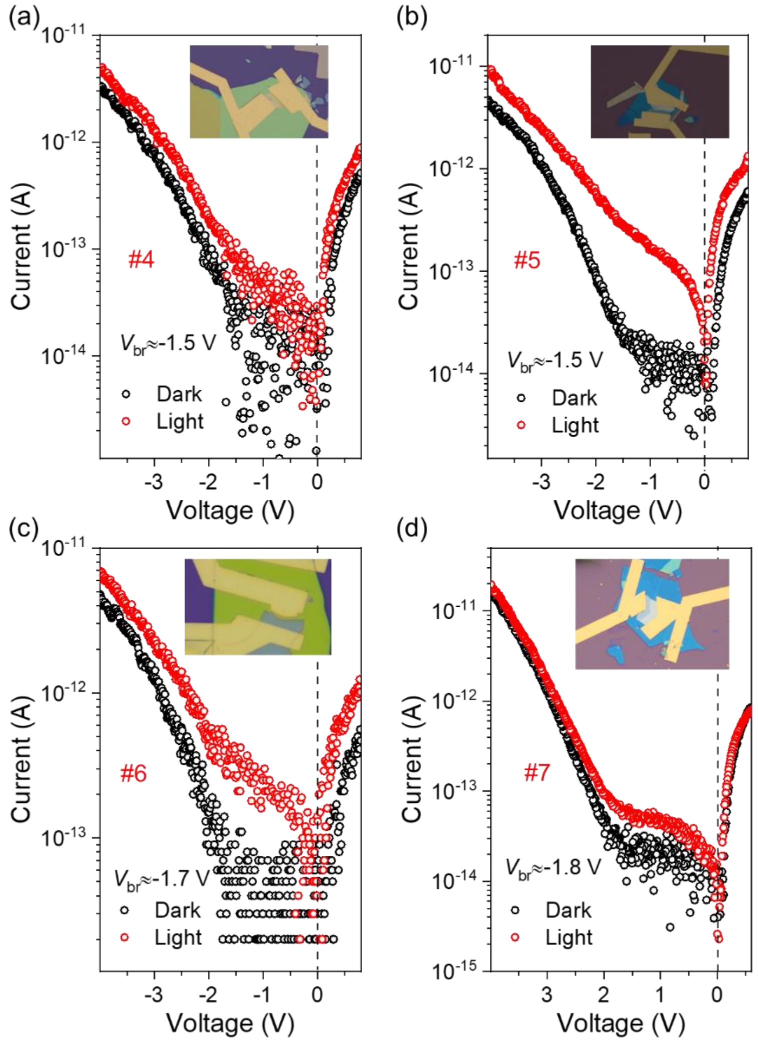

Supplementary Figure 24. *I-V* curves of Numbers (a) #4, (b) #5, (c) #6, and (d) #7 WSe_2 diodes encapsulated with hBN in the dark and under illumination at 300 K. Inset: optical microscope images of the devices.

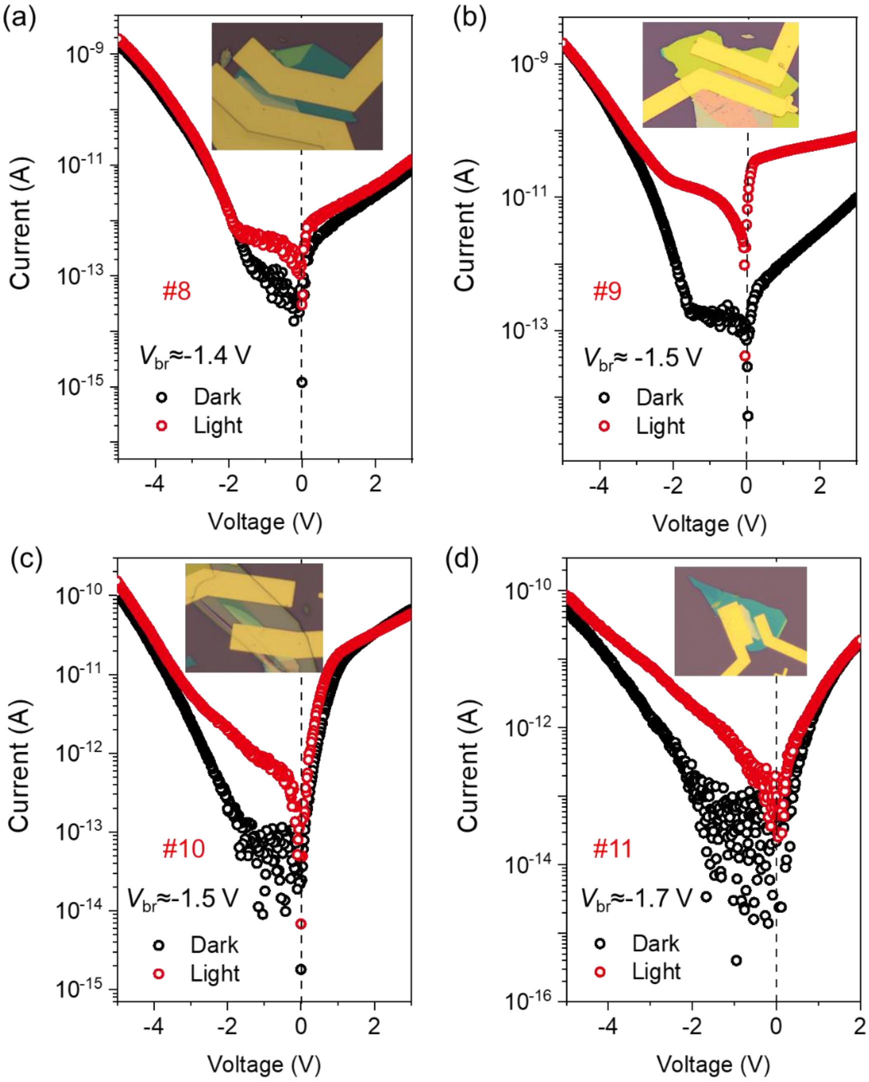

Supplementary Figure 25. *I*-*V* curves of Numbers (a) #8, (b) #9, (c) #10, and (d) #11 WSe_2 diodes encapsulated with BN in the dark and under illumination at 300 K. Inset: optical microscope images of the devices.

Supplementary References

[1] Kang J. et al. Band offsets and heterostructures of two-dimensional semiconductors. *Appl. Phys. Lett.* **102**, 012111 (2013).

[2] Hamamatsu S12023 series, etc. https://www.hamamatsu.com.cn/cn/zh-cn/product/optical-sensors/apd/si-apd/S12023-02.html

[3] Hamamatsu G8931 series. https://www.hamamatsu.com.cn/cn/zh-cn/product/optical-sensors/apd/ingaas-apd/G8931-04.html

[4] Kang Y. et al. Monolithic germanium/silicon avalanche photodiodes with 340 GHz gain–bandwidth product. *Nat. Photon.* **3**, 59–63 (2009).

[5] Verghese S. et al. GaN avalanche photodiodes operating in linear-gain mode and Geiger mode. *IEEE Trans. Electron Devices* **48**, 502-511 (2001).

[6] Ushio PDGAJ Series. https://www.ushio.com/product/pd-ld-germanium-avalanche-photodiode/

[7] Tut T. et al. $Al_xGa_{1-x}N$-based avalanche photodiodes with high reproducible avalanche gain. *Appl. Phys. Lett.* **90**, 163506 (2007).

[8] Meng L. et al. Low-voltage and high-gain WSe_2 avalanche phototransistor with an out-of-plane WSe_2/WS_2 heterojunction. *Nano Res.* **16**, 3422-3428 (2023).

[9] Son B. et al. Efficient Avalanche Photodiodes with a WSe_2/MoS_2 Heterostructure via Two-Photon Absorption. *Nano lett.* **22**, 9516-9522 (2022).

[10] Xia H. Pristine PN junction toward atomic layer devices. *Light: Sci. Appl.* **11**, 170 (2022).

[11] Pak J. et al. Two-dimensional thickness-dependent avalanche breakdown phenomena in MoS_2 field-effect transistors under high electric fields. *ACS Nano* **12**, 7109 (2018).

[12] Gao A. et al. Observation of ballistic avalanche phenomena in nanoscale vertical InSe/BP heterostructures. *Nat. Nanotechnol.* **14**, 217-222 (2019).
